# Supplementary material for: Mast cells interact directly with colorectal cancer cells to promote epithelial-to-mesenchymal transition
Source: Oncogene. 2025 Oct 2;44(45):4391–404. doi: 10.1038/s41388-025-03589-5 (PMC12583129; doi:10.1038/s41388-025-03589-5)
Supplement: Supplementary file 1 — Supplementary Figures S1, S2, S3, S4, S5, and S6 [file 41388_2025_3589_MOESM1_ESM.docx]

**Supplementary Figures**
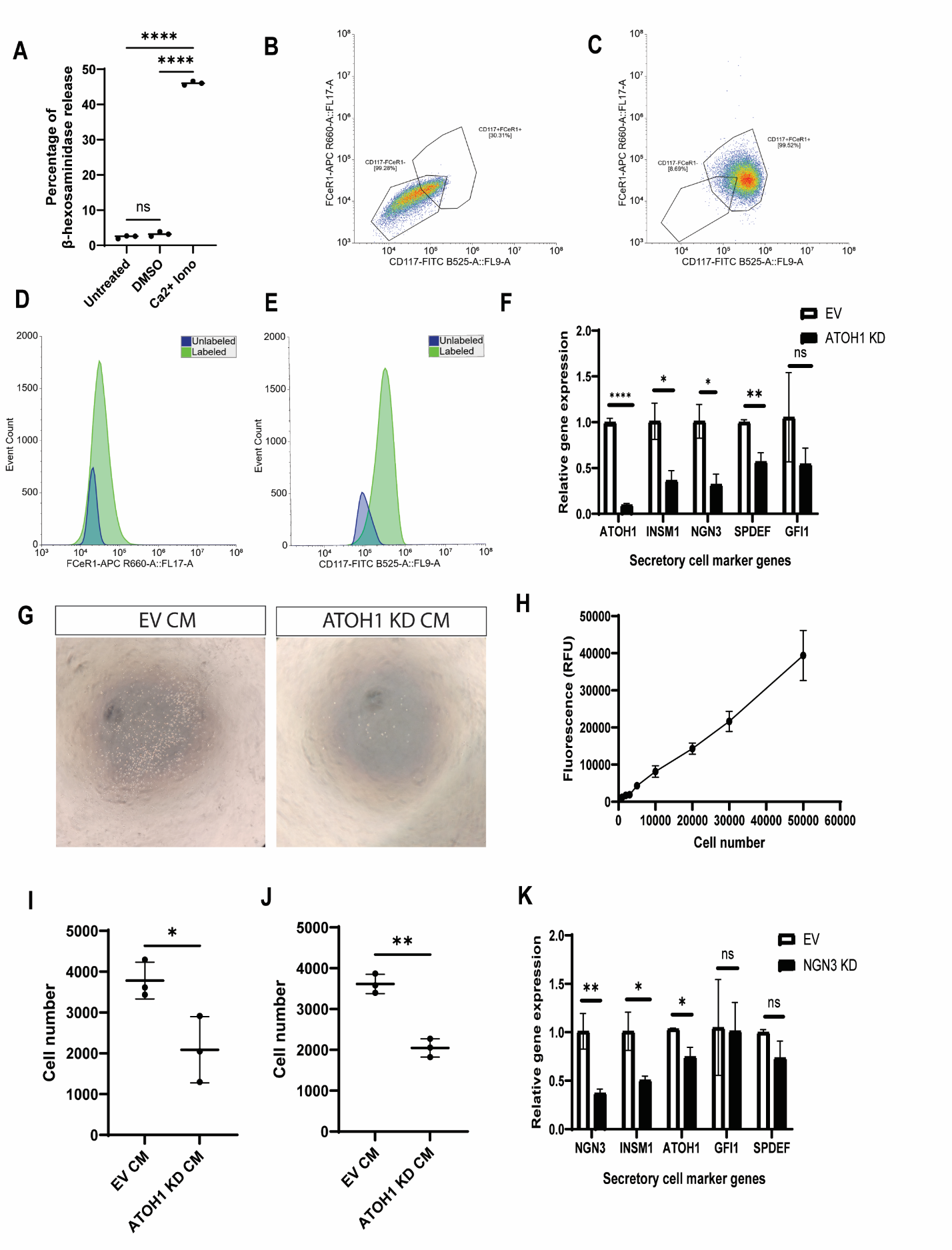

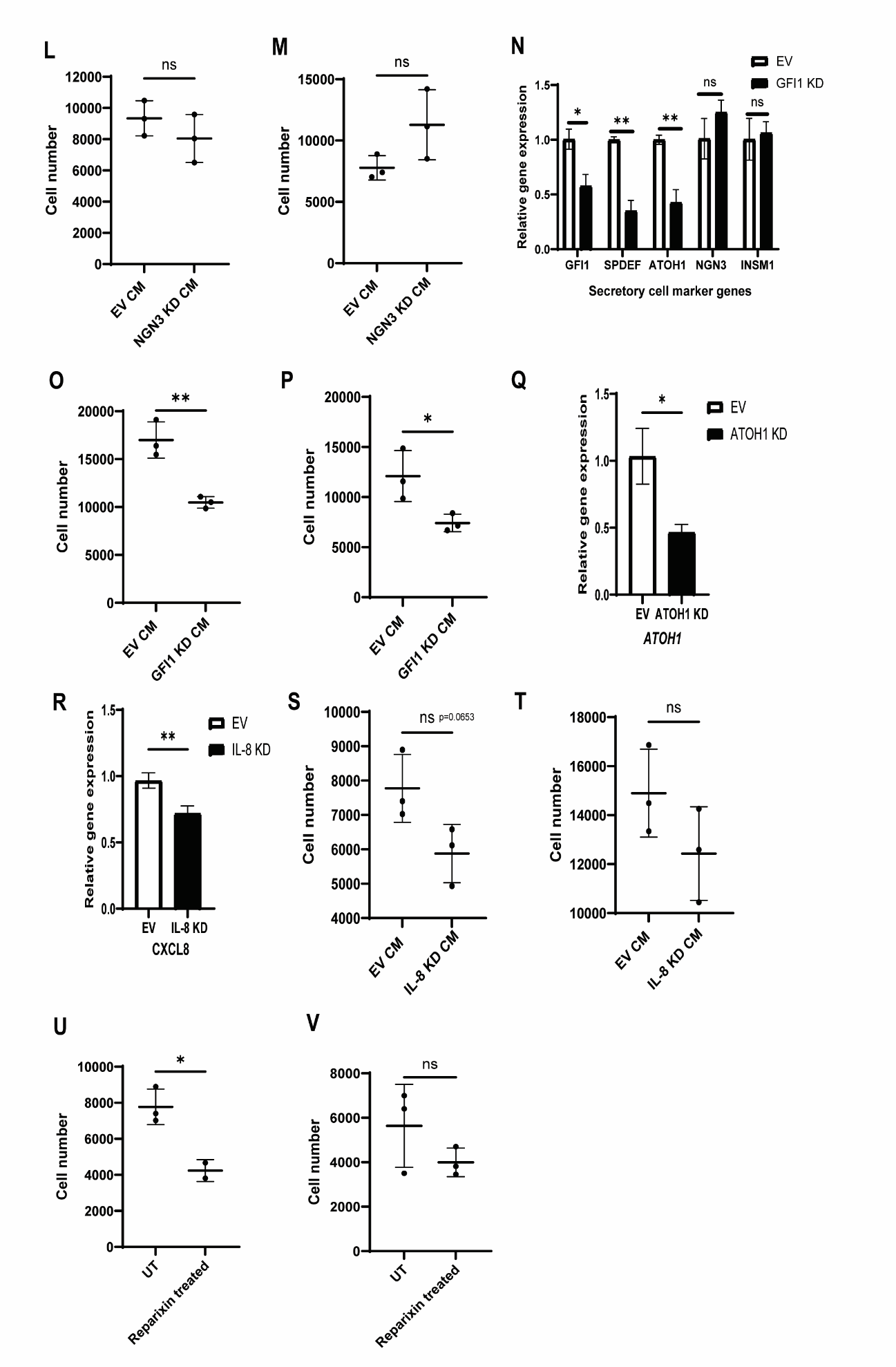


**Supplementary Fig. S1. BMMC differentiation and knockdown confirmation. A**. Percentage of B-hexosaminidase released from untreated, DMSO-treated, and Calcium ionophore (Ca2+ Ionophore, 2 µM, 1h) treated BMMCs. Line indicates the mean, and each point represents an independent biological replicate. **B**. Scatter plot of unlabeled BMMCs analyzed by flow cytometry. **C**. Scatter plot of labeled BMMCs: Cells were labeled with CD117-FITC and FCeR1-APC antibodies and analyzed by flow cytometry. **D**. Histogram representing the distribution of FCeR1-APC signal intensity from gated events in B and C. Data were gated based on forward and side scatter to include single, viable cells only. **E**. Histogram representing the distribution of CD177-FITC signal intensity from gated events in B and C. Data were gated based on forward and side scatter to include single, viable cells only. **F.** Relative qRT-PCR of secretory cell marker genes in empty vector (EV) and ATOH1 knockdown (KD) HT-29 cells. **G**. Images of the lower chamber with migrated BMMCs toward EV or ATOH1 KD conditioned media (CM). **H**. Standard curve of fluorescence in relative fluorescence unit (RFU) as a function of cell numbers. **I, J**. Migration of BMMCs toward CM of EV or ATOH1 KD HT-29 cells. **K.** Relative qRT-PCR of secretory cell marker genes in EV and NGN3 KD HT-29 cells. **L, M**. Migration of BMMCs toward CM of EV or NGN3 KD HT-29 cells. **N**. Relative qRT-PCR of secretory cell marker genes in EV and GFI1 KD HT-29 cells. **O, P.** Migration of BMMCs toward CM of EV or GFI1 KD HT-29 cells. **Q**. Relative qRT-PCR of *ATOH1* in EV and ATOH1 KD 817 organoids. **R**. Relative qRT-PCR of *CXCL8* in EV and IL-8 KD HT-29 cells. **S, T.** Migration of BMMCs toward CM of EV or IL-8 KD HT-29 cells. **U, V.** Migration assay of untreated (UT) or Reparixin-treated (10 µM, overnight) BMMCs toward HT-29 CM. For all panels, graphs indicate mean +/- SD. Significance was determined by two-tailed t-test (I, J, L, M, O, P, Q, R, S, T, U) and one-way ANOVA (A, F, K, N), **p* ≤ 0.05; **p ≤ 0.01; ***p≤ 0.001; ****p≤ 0.0001, ns- not significant.


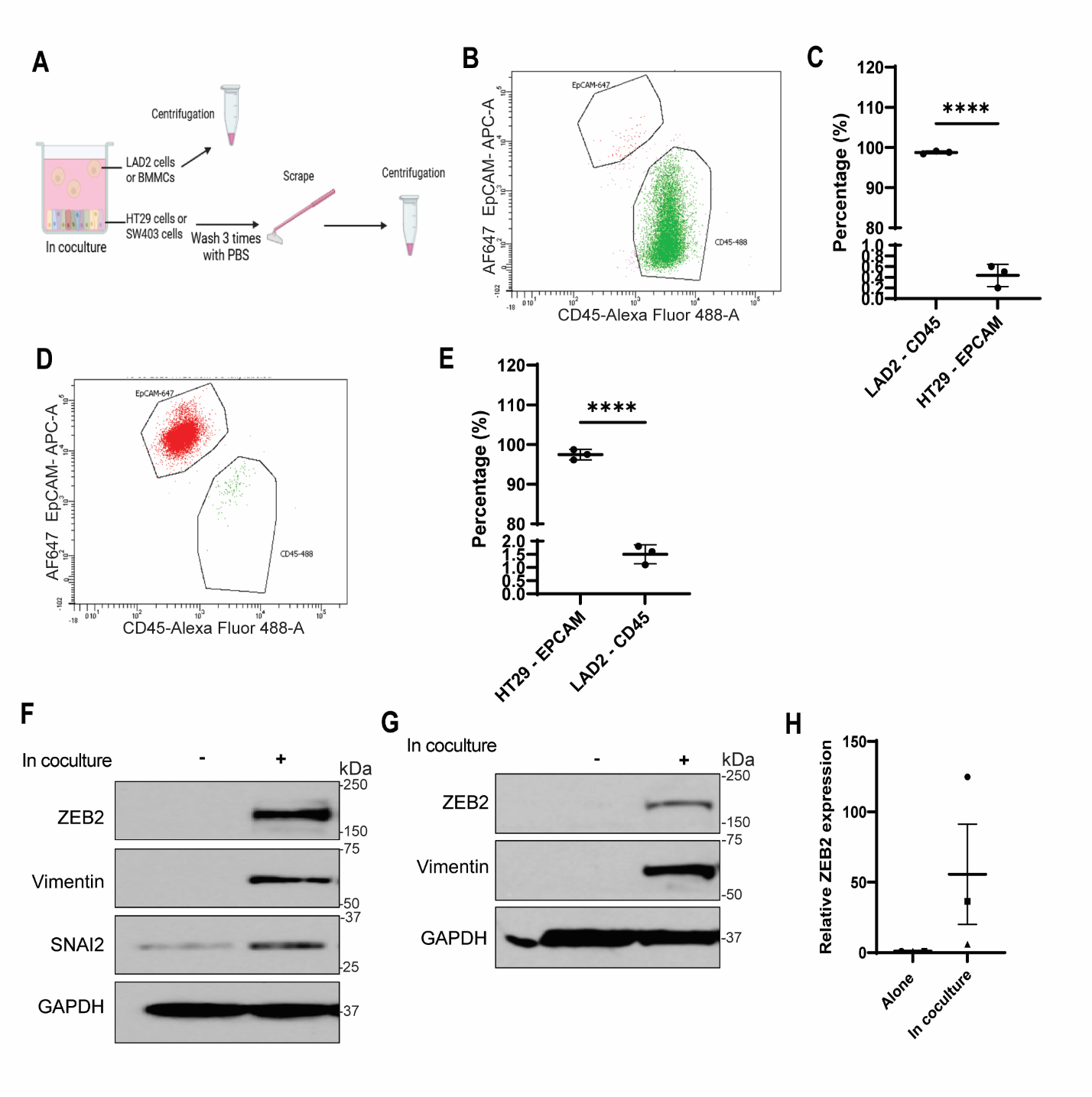


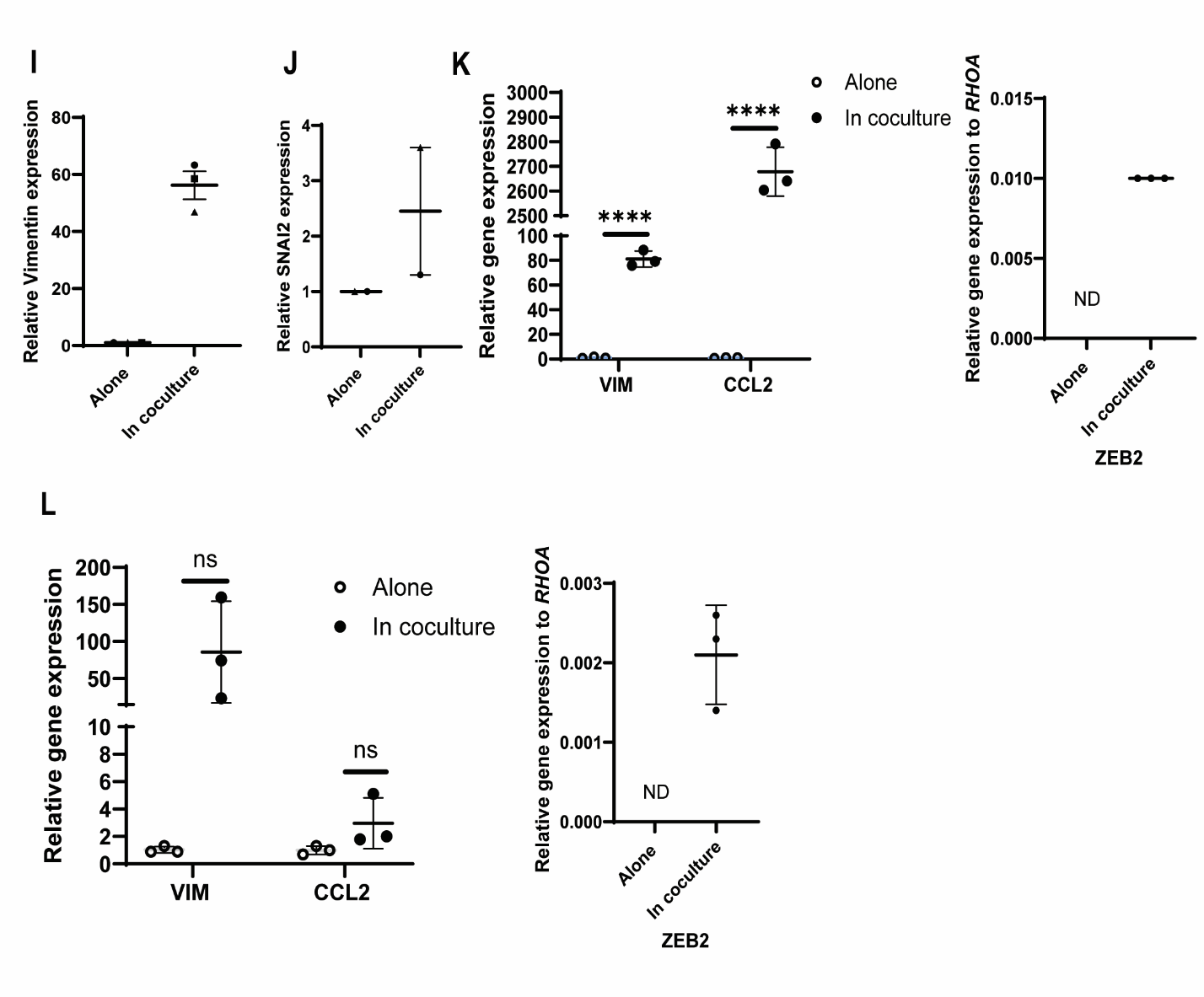


**Supplementary Fig S2. LAD2 and HT-29 cell fractions are 99% and 98% pure after coculture experiments. A**. Scheme of the experimental setup of coculture experiments. **B**. Scatter plot of “LAD2 cell fraction”: Cells were labeled with CD45-AF488 and EPCAM-AF647 antibodies and analyzed by flow cytometry. **C**. Percentage of CD45-AF488 LAD2 cells and EpCam-AF647 HT-29 cells in “LAD2 cell fraction”. N=3. **D**. Scatter plot of “HT-29 cell fraction”: cells were labeled and analyzed as in B. **E**. Percentage of CD45-AF488 LAD2 cells and EpCam-AF647 HT-29 cells in “HT-29 cell fraction”. N=3. **F, G**. Additional western blot replicates of HT-29 cells cocultured alone or with LAD2 cells for 3h. Non-relevant lanes were removed from the blots. **H, I, J**. Relative ZEB2, Vimentin, and SNAI2 expression was normalized to a loading control. Band intensities were quantified using ImageJ and expressed as relative expression to control. Data represent the mean ± SEM from three independent biological replicates (N=3). Symbols represent data points from the same experiment. **K**. Relative qRT-PCR of EMT-related markers and *CCL2* (left) and *ZEB2* (right, not detected (ND)) in HT-29 cells cultured alone or in coculture with BMMCs for 6h. N=3. **L**. Relative qRT-PCR of EMT-related markers and *CCL2* (left) and *ZEB2* (right, not detected (ND)) in SW403 cells cultured alone or in coculture with LAD2 cells for 6h. N=3. For all panels, lines indicate mean +/- SD and each point represents an independent biological replicate. Significance was determined by two-tailed t-test, **p* ≤ 0.05; **p ≤ 0.01; ***p≤ 0.001; ****p≤ 0.0001, ns- not significant.


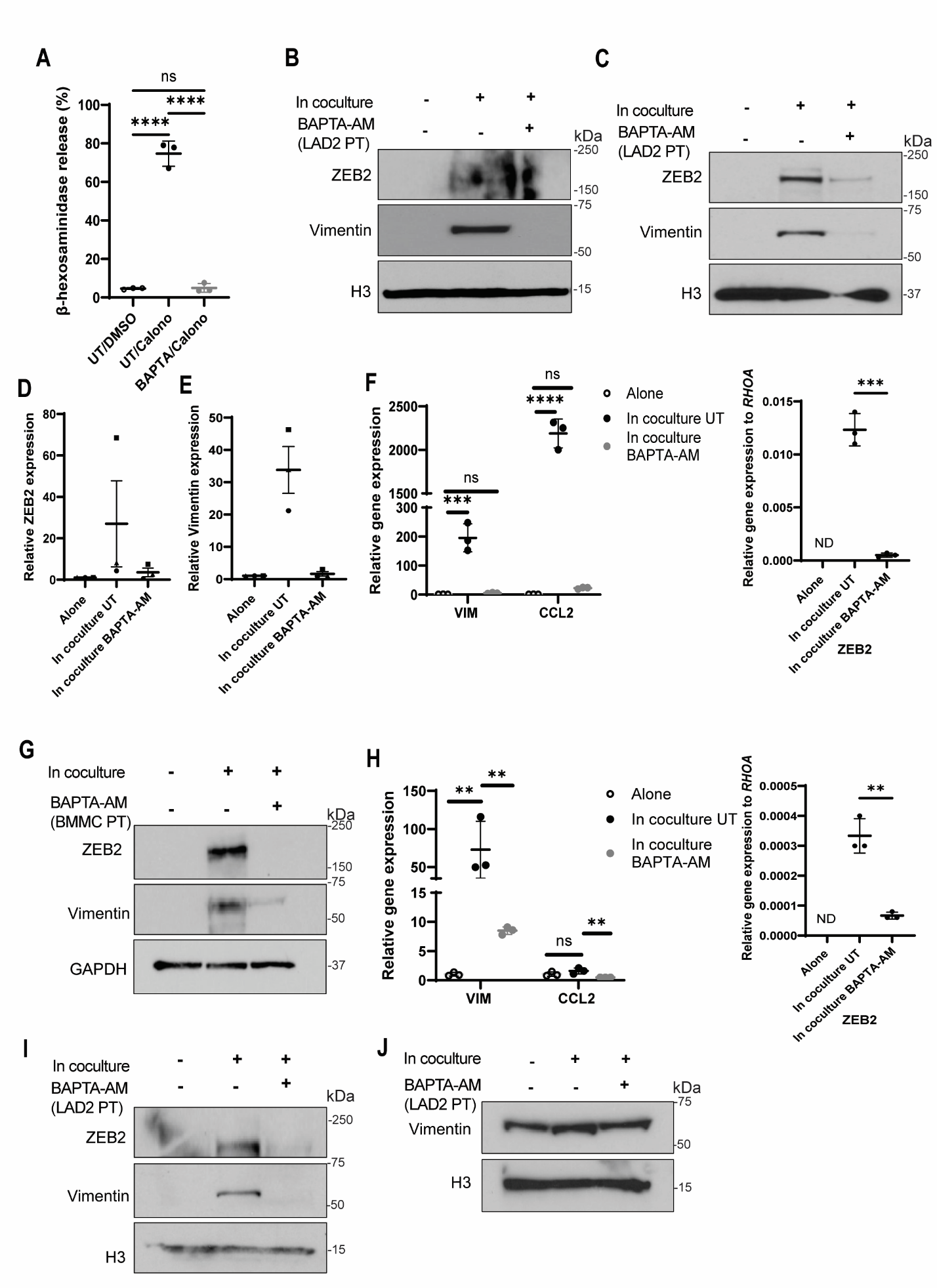


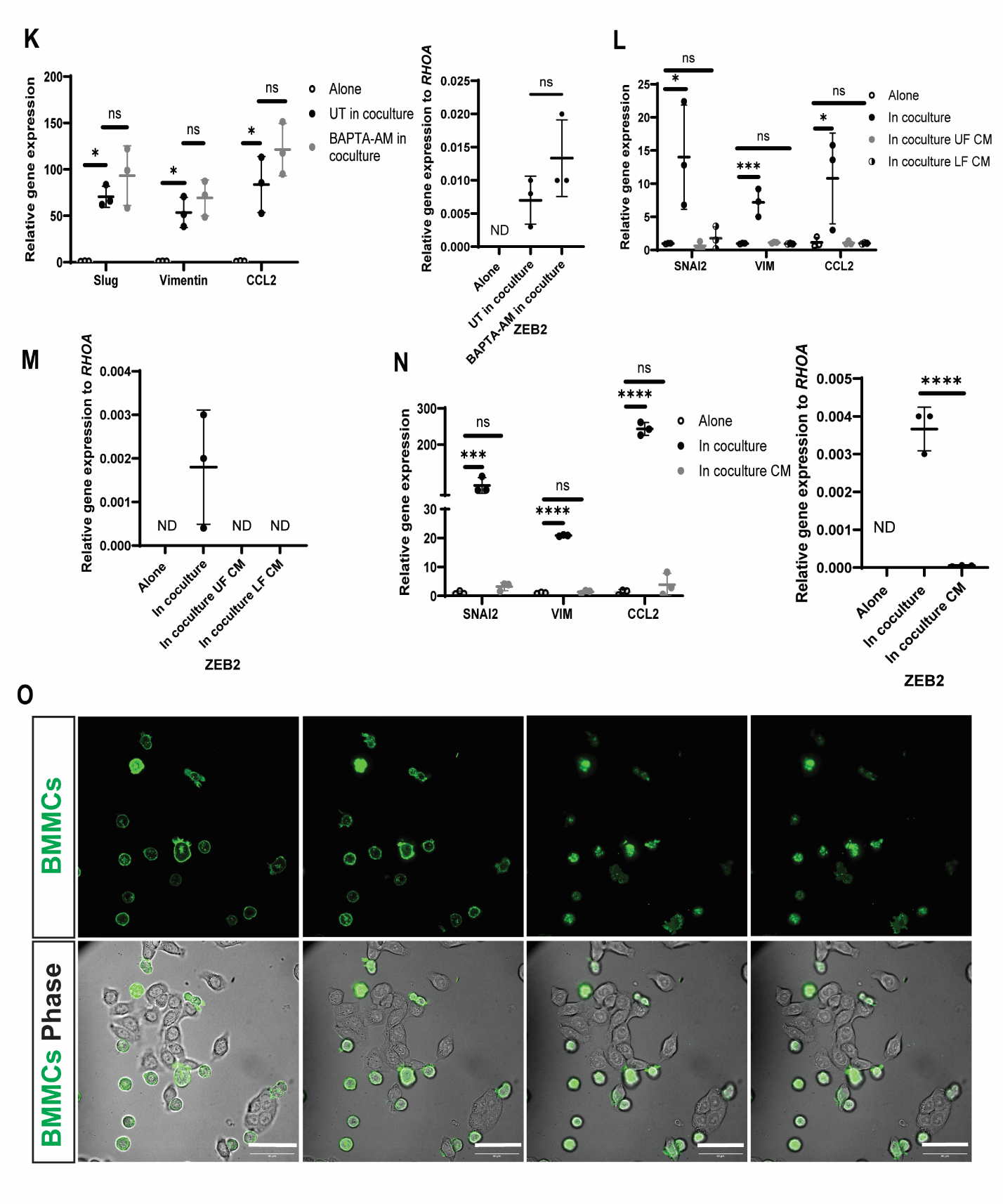


**Supplementary Fig. S3. EMT-related marker expression is calcium and contact-dependent in additional CRC cell and MC lines. A**. Percentage of B-hexosaminidase released in LAD2 cells untreated (UT)/DMSO, untreated in response to calcium ionophore (CaIono; 2 µM, 1h) and pretreated with BAPTA-AM (20 µM, 1h) in response to calcium ionophore. N=1. **B, C**. Additional western blot replicates of HT-29 cells alone, in coculture with unpretreated LAD2 cells, or in coculture with BAPTA-AM (20 µM, 1h) pretreated LAD2 cells (LAD2 PT) for 3h. **D, E**. Relative ZEB2 and Vimentin expression was normalized to a loading control. Band intensities were quantified using ImageJ and expressed as relative expression to control. Symbols represent data points from the same experiment. Data represent the mean ± SEM from three independent biological replicates (N=3). **F**. Relative qRT-PCR of EMT-related marker genes and *CCL2* (left) and *ZEB2* (right, not detected (ND)) in HT-29 cells alone, in coculture with un-pretreated BMMCs, or in coculture with BAPTA-AM (20 µM, for 1h) pretreated BMMCs for 6h. **G**. Western Blot of HT-29 cells alone, in coculture with unpretreated BMMCs, or in coculture with BAPTA-AM pretreated (20 µM, for 1h) BMMCs (BMMC PT) for 3h. N=1. **H**. Relative qRT-PCR of EMT marker genes and *CCL2* (left) and *ZEB2* (right, not detected (ND)) in SW403 cells alone, in coculture with unpretreated LAD2 cells, or in coculture with BAPTA-AM (20 µM, for 1h) pretreated LAD2 cells for 6h. **I**. Western Blot of SW403 cells alone, in coculture with unpretreated LAD2 cells, or in coculture with BAPTA-AM (20 µM, for 1h) pretreated LAD2 cells for 3h. N=2. **J**. Western blot of LAD2 cells alone, untreated in coculture with HT-29 cells, or BAPTA-AM (20 µM, 1h) pretreated (PT) in coculture with HT-29 cells for 3h. N=3. **K**. Relative qRT-PCR of EMT-related marker genes and *CCL2* (left) and *ZEB2* (right, not detected (ND)) in HT-29 cells alone, untreated in coculture with LAD2 cells, or pretreated with BAPTA-AM (20 µM, for 1h) in coculture with LAD2 cells for 6h. **L, M**. Relative qRT-PCR of EMT marker genes and *CCL2* (H) and *ZEB2* (I, not detected (ND)) in HT-29 cells alone, in coculture with LAD2 cells, in concentrated upper fraction of CM (UF CM), or in concentrated lower fraction CM (LF CM) for 6h. **N**. Relative qRT-PCR of EMT marker genes and *CCL2* (left) and *ZEB2* (right, not detected (ND)) in HT-29 cells alone, in coculture with LAD2 cells, or in coculture conditioned media (CM) for 6h. **O**. BMMCs were labeled with DiO (10 µg/ml, 20 minutes, green) and incubated in direct coculture with HT-29 cells (unlabeled). Each panel represents a different focal plane to better demonstrate cell-cell interactions. Live cells were imaged with Olympus OSR SD confocal microscope for 1h. Magnification 60x. Scale bar is 50 µM. Second picture of panel was also used for Figure 4D. For all panels, lines indicate mean +/- SD and each point represents an independent biological replicate. Significance was determined by two-tailed t-test (F right, H right, K right, N right) and one-way ANOVA (A, F left, H left, K left, N left), **p* ≤ 0.05; **p ≤ 0.01; ***p≤ 0.001; ****p≤ 0.0001, ns- not significant.

**Supplementary Video V1. MCs directly interact with CRC cells.** BMMCs were labeled with DiO (10 µg/ml, 20 minutes, green) and incubated in direct coculture with HT-29 cells (unlabeled). Live cells were imaged with Olympus OSR SD confocal microscope. Magnification 60x. Scale bar = 50 µM.


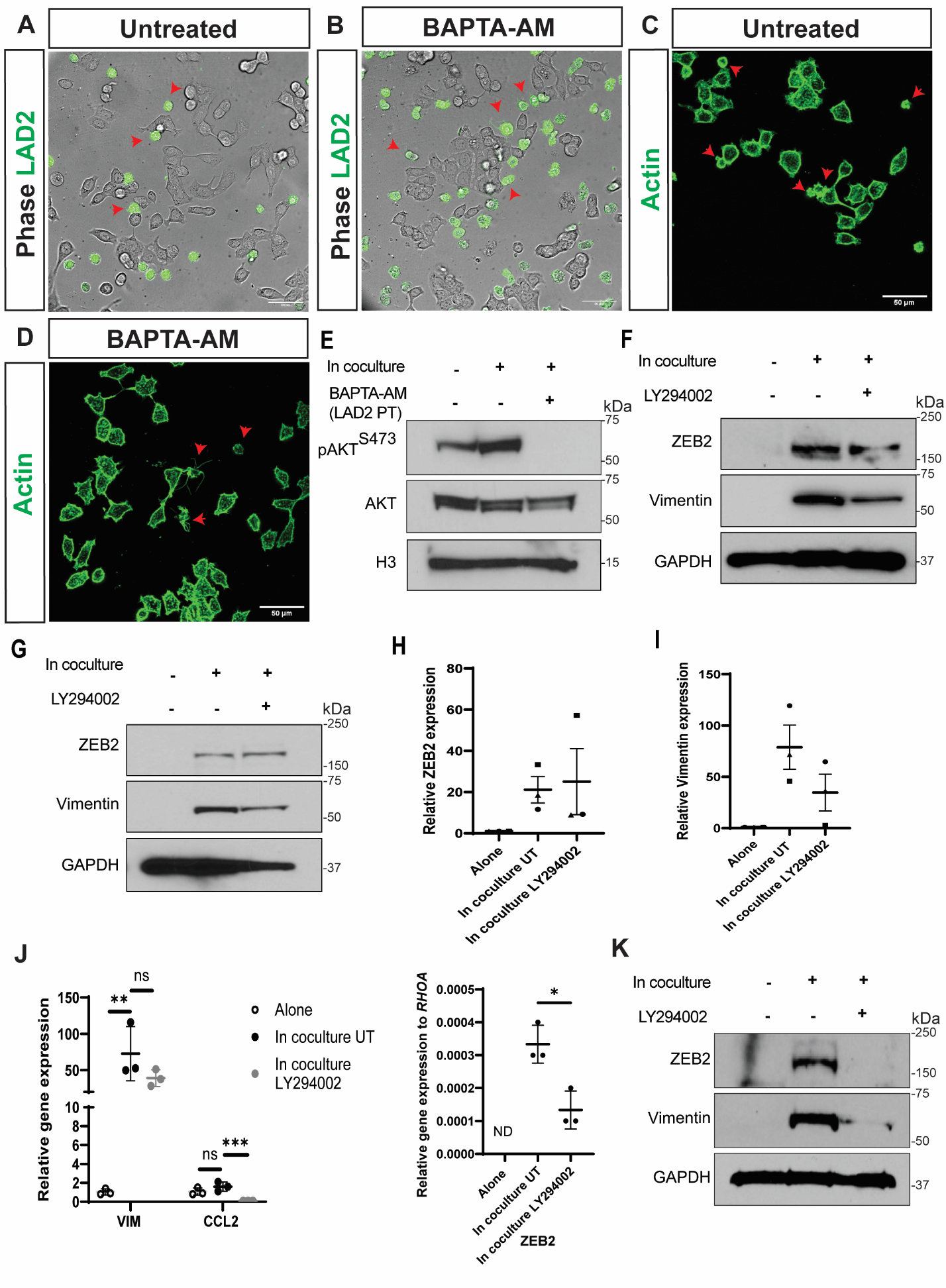

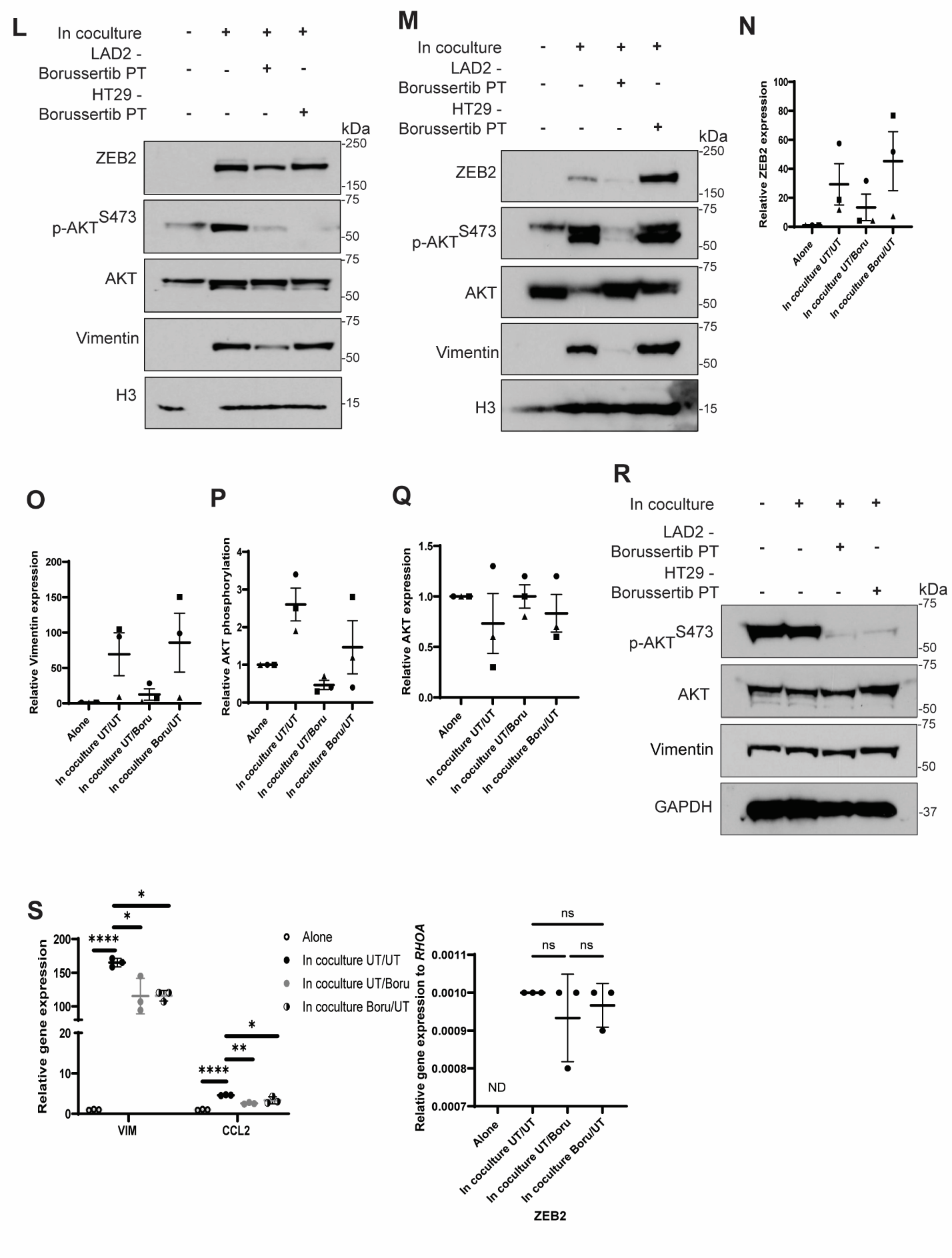


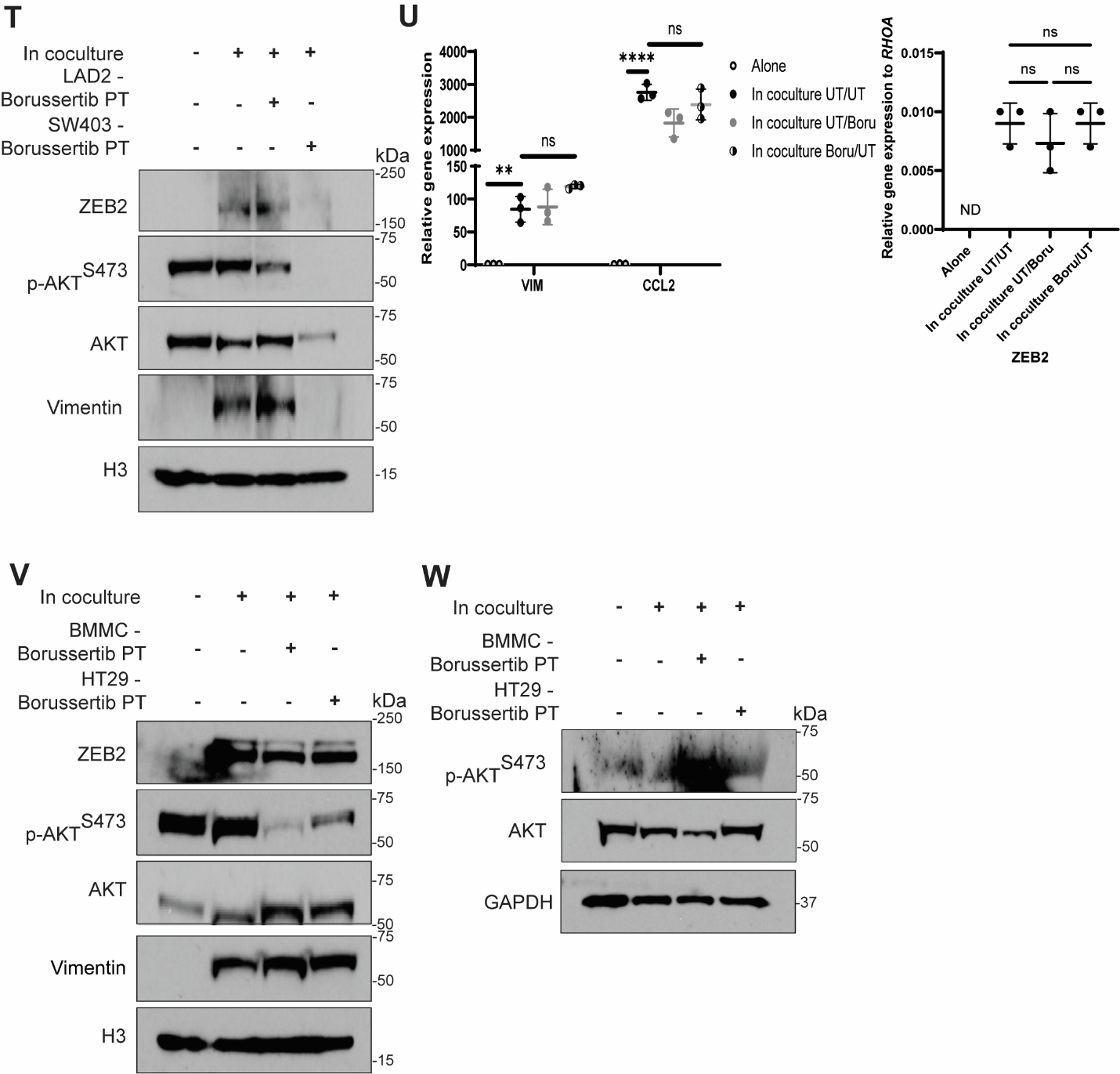


**Supplementary Fig. S4. Role of AKT activation in the induction in EMT-related marker expression in CRC cell lines by MCs. A, B**. Untreated LAD2 cells (A) or BAPTA-AM (20 µM, 1h) pretreated LAD2 cells (B) were labeled with DiO (10 µg/ml, 20 minutes, green) and incubated in direct coculture with HT-29 cells (unlabeled). Live cells were imaged with Olympus OSR SD confocal microscope for 3h. Magnification 40x. **C, D**. Untreated LAD2 cells (C) or BAPTA-AM (20 µM, 1h) pretreated LAD2 cells (D) were incubated in direct coculture with HT-29 cells. Actin cytoskeleton was labeled with Phalloidin-488 reagent. Live cells were imaged with Leica Stellaris 8 FALCON confocal microscope. Magnification 63x. **E**. Western blot of LAD2 cells alone, untreated in coculture with HT-29 cells, or BAPTA-AM (20 µM, 1h) pretreated (PT) in coculture with HT-29 cells for 3h. Western blots for Supplementary Figure S4E and S3H originate from the same blot and have the same loading control. N=3. **F, G**. Additional western blot replicates of HT-29 cells alone, in coculture with untreated (UT) LAD2 cells, or in coculture with LAD2 cells under LY294002 treatment (50 µM). **H, I**. Relative ZEB2 and Vimentin expression was normalized to a loading control. Band intensities were quantified using ImageJ and expressed as relative expression to control. Symbols represent data points from the same experiment. Data represent the mean ± SEM from three independent biological replicates (N=3). **J**. Relative qRT-PCR of EMT related genes and *CCL2* (left) and *ZEB2* (right, not detected (ND)) in SW403 cells alone, in coculture with untreated LAD2 cells, or in coculture with LAD2 cells under LY294002 treatment (60 µM) for 6h. **K**. Western blot of SW403 cells treated and in cocultured as in J for 3h. N=1. **L**, **M.** Additional western blot replicates of HT-29 cells alone, unpretreated and in coculture with unpretreated LAD2 cells, unpretreated and in coculture with Borussertib (1 µM, overnight) pretreated (PT) LAD2 cells, or Borussertib (10 µM, overnight) pretreated in coculture with unpretreated LAD2 cells, HT-29 – Borussertib PT = Borussertib pretreated HT-29 cells. LAD2 – Borussertib PT = Borussertib pretreated LAD2 cells. **N, O, P, Q.** Relative ZEB2, Vimentin expression, AKT phosphorylation, and AKT expression were normalized to a loading control. Band intensities were quantified using ImageJ and expressed as relative expression to control. Symbols represent data points from the same experiment. Data represent the mean ± SEM from three independent biological replicates (N=3). **R.** Western blot of LAD2 cells unpretreated and alone, unpretreated and in coculture with unpretreated HT-29 cells, Borussertib (1 µM, overnight) pretreated in coculture with unpretreated HT-29 cells (LAD2 – Borussertib PT), unpretreated in coculture with Borussertib (10 µM, overnight) pretreated HT-29 cells (HT-29 – Borussertib PT) for 3h. N=3. **S**. Relative qRT-PCR of EMT related genes and *CCL2* (left) and *ZEB2* (right, not detected (ND)) in SW403 cells alone, unpretreated (UT) in coculture with unpretreated LAD2 cells (UT/UT), or unpretreated in coculture with Borussertib (1 µM, overnight) pretreated LAD2 cells (UT/Boru), or Borussertib (10 µM, 1h) pretreated in coculture with unpretreated LAD2 cells (Boru/UT) for 6h. **T**. Western blot of SW403 cells treated and cocultured as in S. N=2. **U**. Relative qRTP-PCR of EMT related genes and *CCL2* (left) and *ZEB2* (right, not detected (ND)) in HT-29 cells unpretreated and alone, unpretreated and in coculture with unpretreated BMMCs (UT/UT), unpretreated in coculture with Borussertib (1 µM, overnight) pretreated BMMCs (UT/Boru), Borussertib (10 µM, overnight) pretreated in coculture with unpretreated BMMCs (Boru/UT) for 6h. **V**. Western blot of HT-29 cells treated and cocultured as in U for 3h. N=1. **W**. Western blot of BMMCs treated and cocultured as in U. N=1. For all panels, lines indicate mean +/- SD and each point represents an independent biological replicate. Significance was determined by two-tailed t-test (J right) and one-way ANOVA (J left, S, U), **p* ≤ 0.05; **p ≤ 0.01; ***p≤ 0.001; ****p≤ 0.0001, ns- not significant.


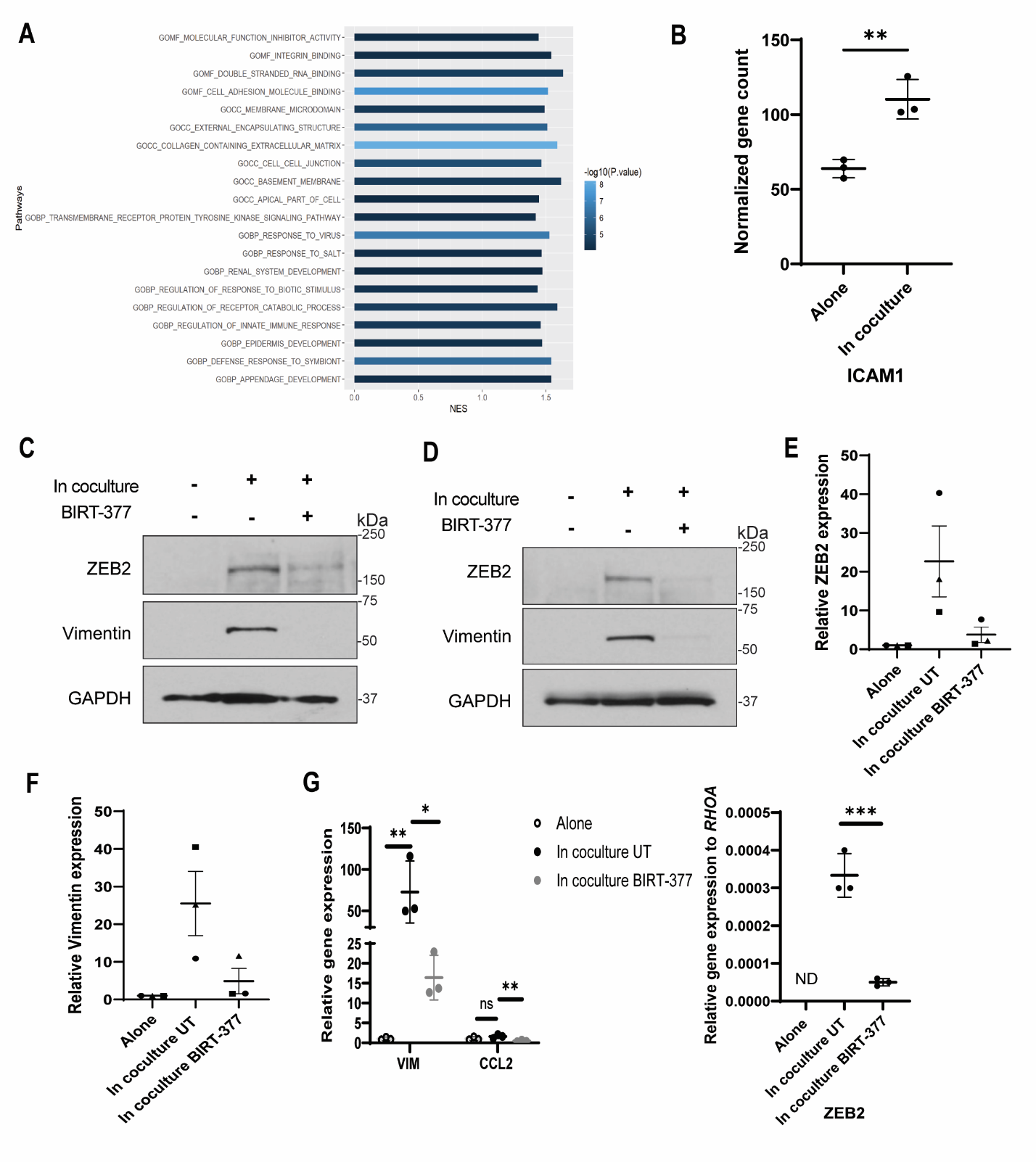


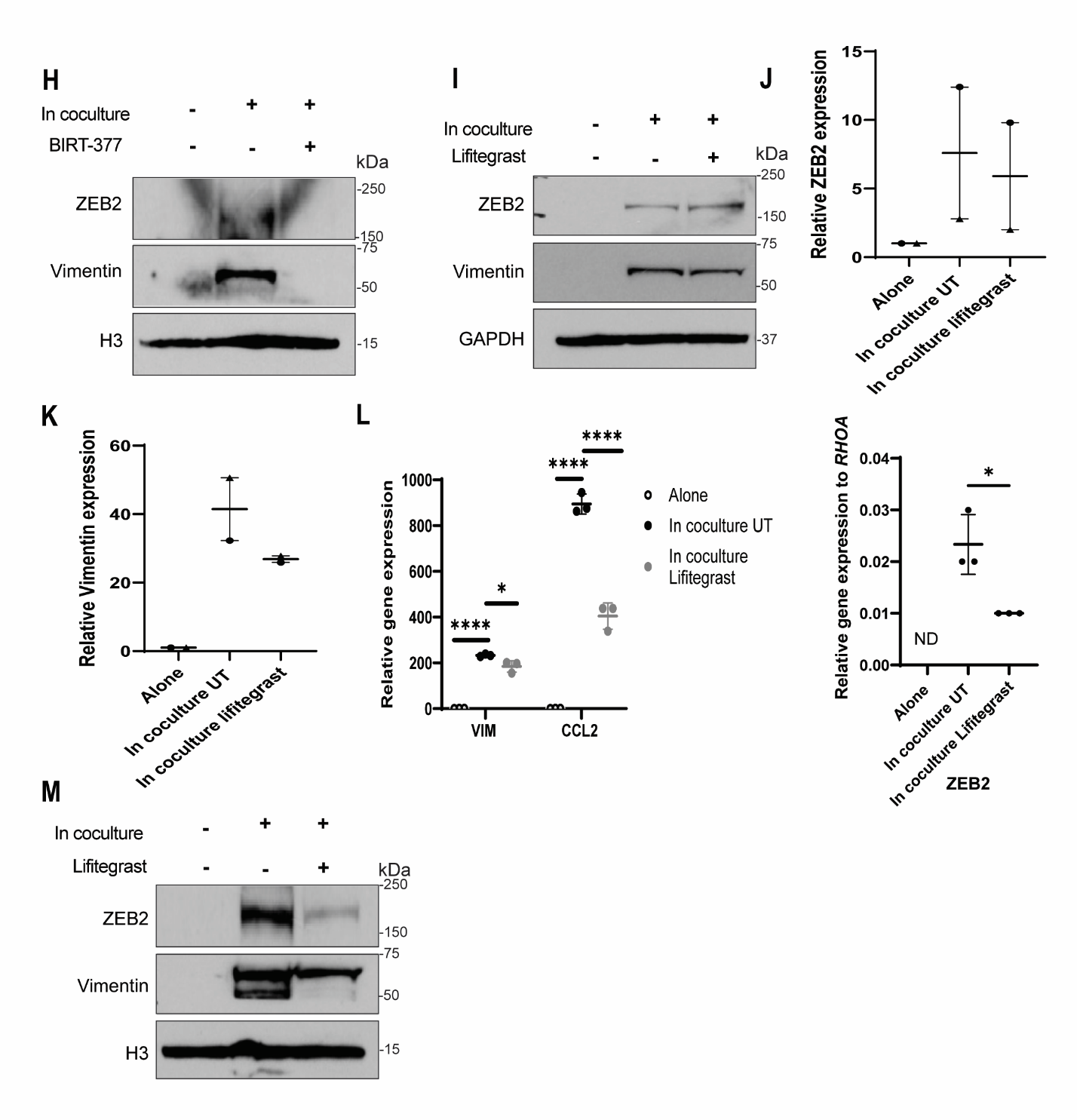


**Supplementary Fig. S5. LFA-1/ICAM-1 integrins are involved in the induction of the EMT-related marker expression in additional CRC cells. A**. Gene ontology enrichment of RNA-sequencing data from LAD2 cells cocultured with HT-29 cells for 12h. **B**. Normalized gene count of *ICAM1* gene from RNA-sequencing data from empty vector HT-29 cells alone or in coculture with LAD2 cells for 12h. **C,D**. Additional western blot replicates of HT-29 cells alone, in coculture with untreated (UT) LAD2 cells, or in coculture with LAD2 cells under BIRT-377 treatment (40 µM) for 6h. **E, F**. Relative ZEB2 and Vimentin expression were normalized to a loading control. Band intensities were quantified using ImageJ and expressed as relative expression to control. Symbols represent data points from the same experiment. Data represent the mean ± SEM from three independent biological replicates (N=3). **G**. Relative qRT-PCR of EMT related genes and *CCL2* (left) and *ZEB2* (right, not detected (ND)) in SW403 cells alone, in coculture with untreated (UT) LAD2 cells, or in coculture with LAD2 cells under BIRT-377 treatment (in coculture BIRT-377) (40 µM) for 6h. **H**. Western blot of SW403 cells treated and cocultured as in G for 3h. N=1. **I**. Additional western blot replicate of HT-29 cells alone, in coculture with untreated (UT) LAD2 cells, or in coculture with LAD2 cells under Lifitegrast treatment (40 µM) for 6h. **J, K**. Relative ZEB2 and Vimentin expression were normalized to a loading control. Band intensities were quantified using ImageJ and expressed as relative expression to control. Data represent the mean ± SEM from three independent biological replicates (N=3). **L**. Relative qRT-PCR of EMT related genes and *CCL2* (left) and *ZEB2* (right, not detected (ND)) in HT-29 cells alone, in coculture with untreated (UT) BMMCs, or in coculture with BMMCs under Lifitegrast treatment (40 µM) for 6h. **M**. Western blot of HT-29 cells treated and cocultured as in L for 3h. N=1. For all panels, lines indicate mean +/- SD and each point represents an independent biological replicate. Significance was determined by two-tailed t-test (B, G right, L right) one-way ANOVA (G left, L left), **p* ≤ 0.05; **p ≤ 0.01; ***p≤ 0.001; ****p≤ 0.0001, ns- not significant.


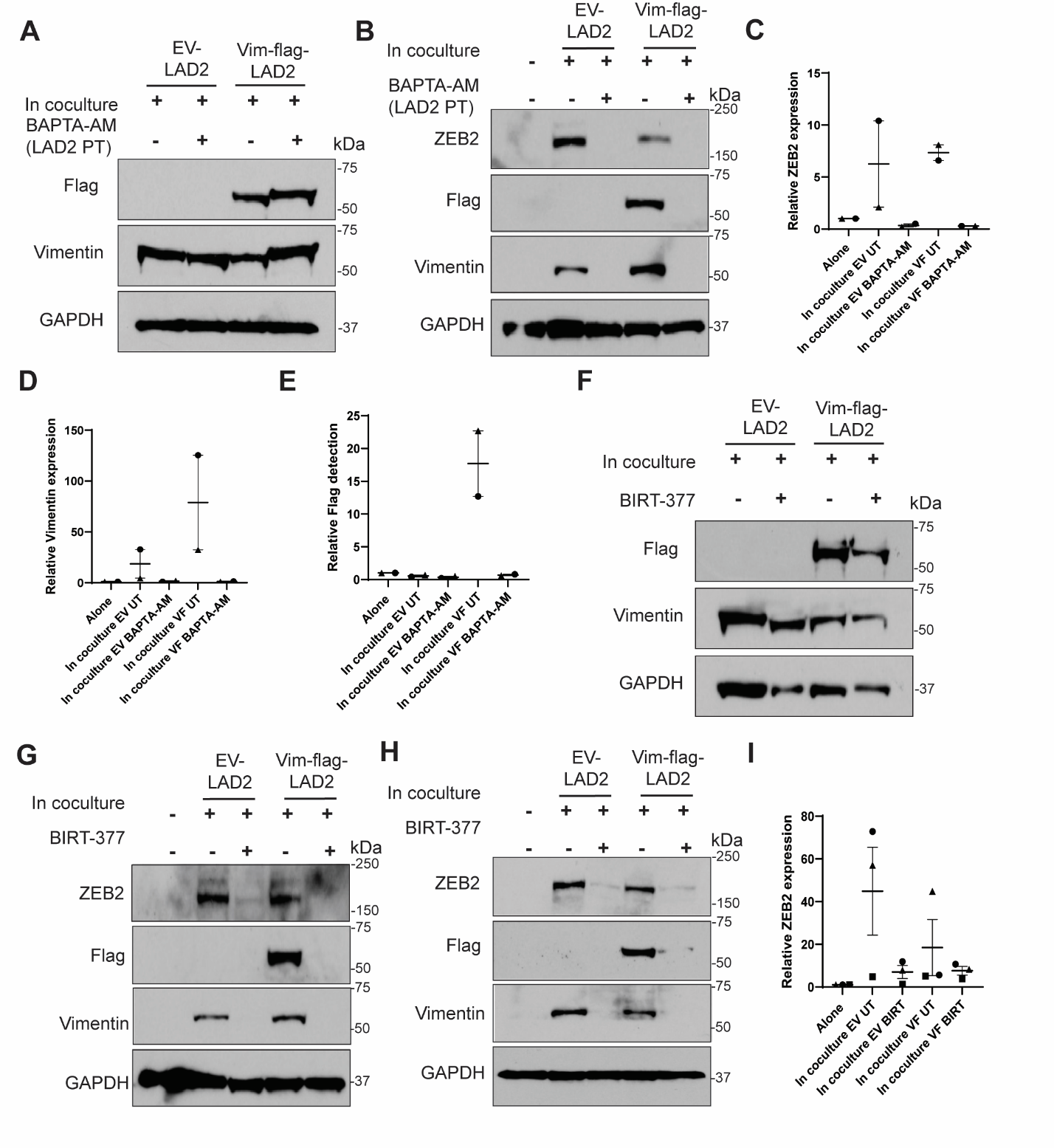


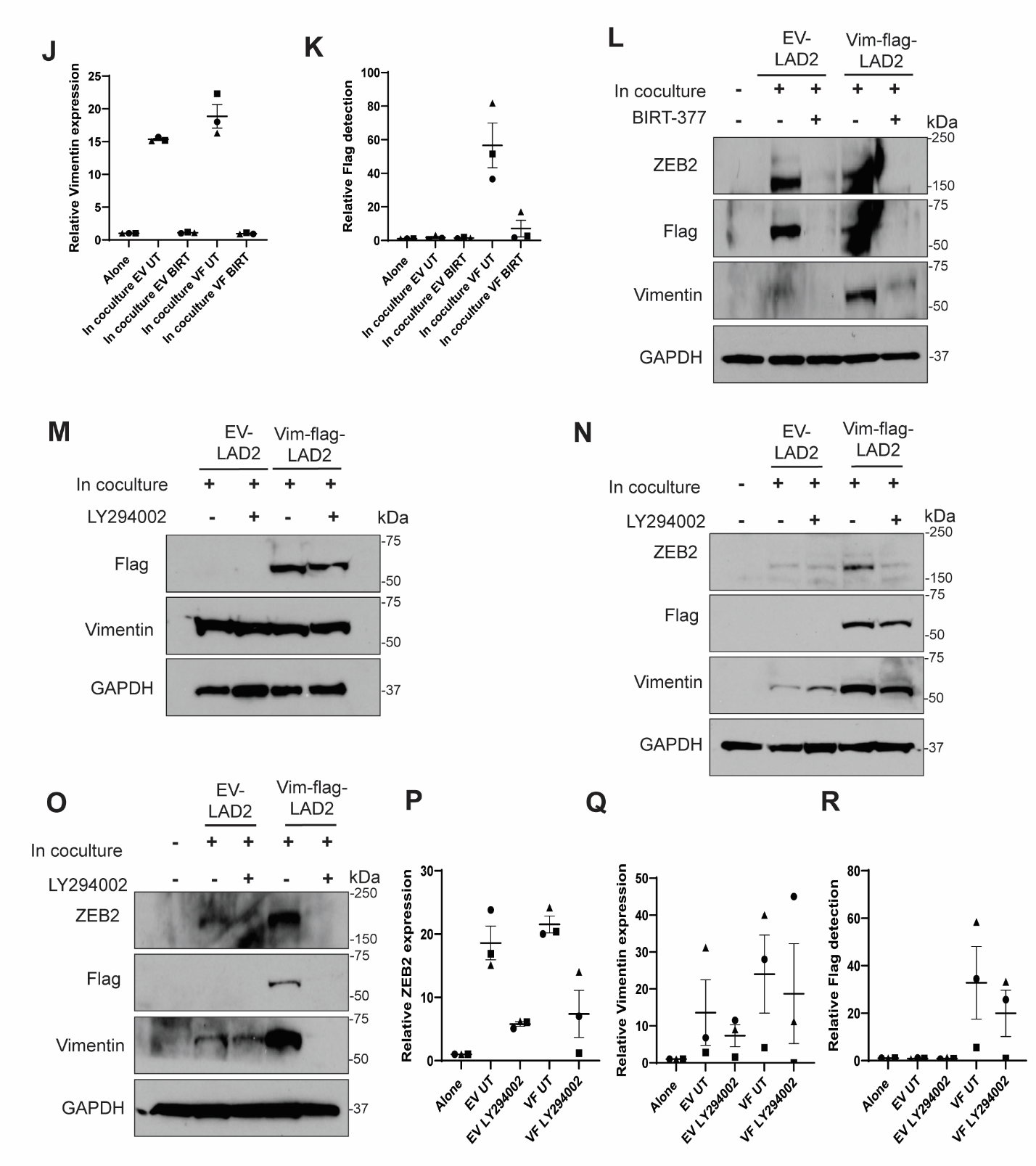


**Supplementary Fig. 6. Treatment does not change Vimentin-Flag expression in MCs. A**. Western blot of unpretreated empty vector (EV-) LAD2 cells in coculture with HT-29 cells, BAPTA-AM (20 µM, 1h) pretreated EV LAD2 cells in coculture with HT-29 cells, unpretreated Vimentin-Flag transduced (Vim-Flag) LAD2 cells with HT-29 cells, or BAPTA-AM (20 µM, 1h) pretreated Vim-Flag LAD2 cells in coculture with HT-29 cells for 3h. N=1. **B**. Additional western blot replicate of HT-29 cells alone or in coculture with unpretreated empty vector (EV-) LAD2 cells, BAPTA-AM (20 µM, 1h) pretreated EV LAD2 cells, unpretreated Vimentin-Flag transduced (Vim-flag-) LAD2 cells, or BAPTA-AM (20 µM, 1h) pretreated Vim-Flag LAD2 cells. **C, D, E**. Relative ZEB2, Vimentin expression, and Flag detection were normalized to a loading control. Band intensities were quantified using ImageJ and expressed as relative expression to control. Symbols represent data points from the same experiment. Data represent the mean ± SEM from two independent biological replicates (N=2). **F**. Western blot of untreated EV LAD2 cells in coculture with HT-29 cells, EV LAD2 cells in coculture with HT-29 cells under BIRT-377 treatment (20 µM), untreated Vim-Flag LAD2 cells in coculture with HT-29 cells, or Vim-Flag LAD2 cells in coculture with HT-29 cells under BIRT-377 treatment (20 µM) for 3h. N=1. **G, H**. Additional western blot replicates of HT-29 cells alone, in coculture with untreated EV LAD2 cells or in coculture with EV LAD2 cells under BIRT-377 treatment (20 µM), untreated Vim-Flag LAD2 cells, or Vim-Flag LAD2 cells under BIRT-377 treatment (20 µM). N=3. **I, J, K**. Relative ZEB2, Vimentin expression, and Flag detection were normalized to a loading control. Band intensities were quantified using ImageJ and expressed as relative expression to control. Symbols represent data points from the same experiment. Data represent the mean ± SEM from three independent biological replicates (N=3). **L**. Western blot of SW403 cells alone, in coculture with untreated EV LAD2 cells, in coculture with EV LAD2 cells under BIRT-377 treatment (40µM), in coculture with untreated Vim-Flag LAD2 cells, or in coculture with Vim-Flag LAD2 cells under BIRT-377 treatment (40µM) for 3h. N=1. **M**. Western blot of untreated EV LAD2 cells in coculture with HT-29 cells, EV LAD2 cells in coculture with HT-29 cells under LY294002 treatment (50 µM), untreated Vim-Flag LAD2 cells in coculture with HT-29 cells, or Vim-Flag LAD2 cells in coculture with HT-29 cells under LY294002 treatment (50 µM) for 3h. N=1. **N, O.** Additional western blot replicates of HT-29 cells alone or in coculture with untreated EV LAD2 cells, EV LAD2 cells under LY294002 treatment (50 µM), untreated Vim-Flag LAD2 cells, or Vim-flag LAD2 cells under LY294002 treatment (50 µM). Blot for ZEB2 expression in S6N has been run on a different membrane. **P, Q, R**. Relative ZEB2, Vimentin expression, and Flag detection were normalized to a loading control. Band intensities were quantified using ImageJ and expressed as relative expression to control. Symbols represent data points from the same experiment. Data represent the mean ± SEM from three independent biological replicates (N=3).
