# Supplementary material for: Mast cells interact directly with colorectal cancer cells to promote epithelial-to-mesenchymal transition
Source: Oncogene. 2025 Oct 2;44(45):4391–404. doi: 10.1038/s41388-025-03589-5 (PMC12583129; doi:10.1038/s41388-025-03589-5)
Supplement: Supplementary file 3 — Supplementary Methods [file 41388_2025_3589_MOESM3_ESM.docx]

**Supplementary Methods**

**β-hexosaminidase release assay**

β-hexosaminidase release assay was measured as described previously (1). LAD2 cells (1 × 10^4 cells/ml) in triplicates were incubated in clear bottom 96 well plate at 37°C in 5% CO2 in Hanks balanced salt solution (Thermo Fischer Scientific, J67799.AP) for 1 h in the presence of DMSO, or 2 µM calcium ionophore A23187. Cells were centrifuged at 600xg for 10 min. Supernatants were incubated with 1 mM p-nitro-phenyl-N-acetyl-β-d-glucosaminide (Cayman chemical, 28954) in 0.05 M citrate buffer (pH 4.5) at 37°C for 1 h. As a control for total β-hexosaminidase content, cells were lysed with 150ul of 1% Triton X-100 and incubated as above. All reactions were quenched by addition of 50 µl of glycine buffer (pH 10.0) and the absorbance was read at 405 nm. The percentage of degranulation can be calculated by % =  100 x (supernatant content)/(supernatant+lysate content) as described previously (2).

**Immunohistochemistry**

Tryptase was detected by IHC on 8-μm FFPE CRC samples following unmasking in citrate buffer. Anti-Tryptase (Cell Signaling Technology #19523) was applied at a dilution of 1:40, followed by rabbit HRP SignalStain Boost (Cell Signaling Technology, #8125) and DAB substrate (CST, #8059). MCPT1 was detected by IHC on 8-μm FFPE mouse colon sections following unmasking in citrate buffer. Anti-MCPT1 (Invitrogen, 14-5503-82) was applied at a dilution of 1:100, followed by rat HRP SignalStain Boost (Cell Signaling Technology, #72838) and DAB substrate (CST, #8059). Slides were counterstained in hematoxylin. Tumors stained for MCPT1 and Tryptase were scored from 0 to 4: 0: no staining, 1:staining on edge of tumor, 2:staining inside tumor < 5% tumor volume, 3: staining inside tumor 5-10% tumor volume, and 4: staining inside tumor > 10% tumor volume. All scoring was done in a blinded manner.

**Organoid culture and knockdown**

817 organoids were maintained in Intesticult organoid growth media (OGM; Stemcell Technologies; 06010) and Matrigel (Corning) following standard protocols (3). For migration assay, 2 days after passaging, media was changed to Intesticult organoid differentiation media (ODM; Stemcell Technologies; 100-0214).

To generate the knockdown of ATOH1 (Sigma-Aldrich, SHCLNG- NM_005172, TRCN0000433098) and empty vector TRC2 (Sigma-Aldrich, #SHC201), the following lentiviral transduction protocol was used. 817 organoids were dissociated to single cells following standard protocol. Cell pellet was resuspended with virus-containing media and polybrene. Cells were spun at 600xg for 1h at 32°C. Cells were then incubated for 3h. Cells were resuspended in media and spun at 300xg for 3 minutes at 4°C. Cells were plated in Matrigel dome, and media was added. Puromycin was added 2 days after transduction. Puromycin-free media was used on the day of the migration.

**Flow cytometry**

LSR II Flow cytometer (BD Biosciences) was used for the analysis of CD45-AF488 (Invitrogen, MA5-38729) and EpCam-AF647 (Invitrogen, MA5-38714) staining. CD45-AF488 cells were measured using 488 nm excitation and signal was detected using AF488 (green). EpCam-AF647 cells were measured using 647 nm excitation and signal was detected using AF647 (red). Cytoflex LX (Beckman Coulter) was used for the analysis of CD117-FITC (Invitrogen, 11-1178-42) and FceR1 alpha-APC (Invitrogen, 17-5899-42) staining. CD117-FITC cells were measured using 488 nm excitation and signal was detected using FITC (green). FceR1 alpha cells were measured using 640 nm excitation and signal was detected using APC (red). Cells were labeled with LIVE/DEAD™ Fixable Violet Dead Cell Stain Kit (Thermo Fischer Scientific, L34963) and signal was measured using 416 nm excitation. Samples with no stain were analyzed for every experiment for compensation control.

**Fixed cell Imaging and actin cytoskeleton labeling**

HT-29 cells were seeded on Poly-D-lysine (Gibco, A3890401) coated on No 1.5 coverslip. The next day, untreated or BAPTA-AM pretreated LAD2 cells were incubated with HT-29 cells for 1 hour. At the end of the incubation, the coculture was washed once carefully with PBS. Cells were fixed in 4% paraformaldehyde for 45 minutes at 37°C. Cells were then washed once with PBS for 5 minutes. Cells were permeabilized with 0.5% Triton-x100 for 5 minutes. 350 µl of 1X Phalloidin conjugate working solution (abcam, ab176753) was added to fixed cells for 90 minutes at room temperature. Images were acquired on a Leica STERLLARIS 8 Falcon scanning confocal system with MDi8-inverted microscope with LASX software (Leica Microsystems). All the images were taken at 63X magnification, 1.2A water immersion at room temperature, and processed using ImageJ.

**Concentrated conditioned media preparation**

Conditioned media from the direct coculture between HT-29 and LAD2 cells were collected, ran through protein concentrator columns (Thermo Fischer Scientific, 88539), and centrifuged at 3000xg for 15 minutes at 4°C. Proteins over 100 kDa were concentrated in the upper fraction. Proteins lighter than 100 kDa were concentrated in the lower fraction. Both fractions were run through protein concentrator columns (Thermo Fischer Scientific, 88515) at 3000xg for 50 minutes at 4°C. Concentrated upper and lower fraction conditioned media were used for the incubation of naïve HT-29 cells.

**Antibodies**

For Western blot, anti-Vimentin (CST, 5741, 1:1000,) anti-Slug (CST, 9585, 1:1000), anti-total AKT (CST, 4691,1:1000), anti-Phospho-AKT(Ser473) (CST, 4060, 1:1000), anti-H3 (CST, 4499, 1:1000), anti-GAPDH (CST, 5174, 1:1000), anti-ZEB2 (CST, 97885, 1:1000), anti-FLAG (Sigma-Aldrich, F1804, 1:1000) antibodies were used.

**qRT-PCR primer sequences**

|  | Forward primer sequence | Reverse primer sequence |
| --- | --- | --- |
| RHOA | CGTTAGTCCACGGTCTGGTC | ACCAGTTTCTTCCGGATGGC |
| NEUROG3 | CTCACCAAGATCGAGACGCT | GTACAAGCTGTGGTCCGCTA |
| ATOH1 | AGAGAGCATCCCGTCTACCC | GCTCCGGGGAATGTAGCAAA |
| GFI1 | CTCGCCCACCTCTTCCAAATTTAAC | GTCACTCCGAGGGCTTGCTC |
| SNAI2 | CTCATCTTTGGGGCGAGTGA | CAATGGCATGGGGGTCTGAA |
| Vimentin | GCTTCAGAGAGAGGAAGCCGAAAA | CCGTGAGGTCAGGCTTGGAAA |
| ZEB2 | CCCTGGCACAACAACGAGAT | AATTGCGGTCTGGATCGTGG |
| CCL2 | AGCCAGATGCAATCAATGCC | AGCTTCTTTGGGACACTTGCT |
| IL8 | ACCGGAAGGAACCATCTCAC | GGCAAAACTGCACCTTCACAC |
| Vimentin-flag | CCCTGAACCTGAGGGAAACT | ACCGTCATGGTCTTTGTAGTCT |
| ICAM1 | CTGGGAACAACCGGAAGGTG | GTTCCACCCGTTCTGGAGTC |

**Knockdown and stable lines**

Generation of stable cell lines: to generate lentivirus for Vimentin-promoter-mCherry plasmid (VectorBuilder), Vimentin-Flag plasmid (VectorBuilder), knockdown of ATOH1 (Sigma-Aldrich, SHCLNG- NM_005172, TRCN0000433098) NEUROG3 (Sigma-Aldrich, SHCLNG- NM_020999, TRCN0000427521), GFI1 (Sigma-Aldrich, SHCLNG-NM_005263, TRCN0000413122), IL8 (Sigma-Aldrich, SHCLNG- NM_000584, TRCN0000058028)  and empty vector TRC2 (Sigma-Aldrich, #SHC201), the following lentiviral protocol was used. Briefly, 3.5x10^5 HEK293T cells were plated on day 1 in DMEM 1X containing 10% FBS. The next day cells were transfected with shRNA of interest, EV control, and packaging plasmids. On the third day, the media was replaced with fresh DMEM with 10% FBS. 24hrs later, media containing lentiviral particles was collected and filtered using a 0.22 µm filter, and concentrated with Lenti-X TM Concentrator (Takara, #631232). Media was replaced and on day 5, the media was again collected, filtered using a 0.22 µm filter, and concentrated with Lenti-X TM Concentrator and combined with the media collected on day 4. To perform the transduction (except for Vim-promoter mCherry construct), virus-containing media and polybrene were added to cells. The next day, to select for transduced cells, cells were treated with puromycin (2µg/mL) (Sigma-Aldrich #P8833). Lentiviral transduction increased baseline expression of Vimentin in HT-29 cells. To bypass that complication, we generated a stable Vimentin-promoter-mCherry line using ThermoFischer Scientific stable transfection protocol. Briefly, HT-29 cells were transfected with 5 µg of Vimentin-promoter-mCherry plasmid DNA combined with Optimem media and Lipofectamine 3000 (Invitrogen, L3000001). Puromycin (2 µg/ml) was added to experimental cells 24 hours later. Surviving cells were passaged 48 hours after the addition of puromycin. 24 hours after passaging, puromycin was added back to the media to select for transfected cells.

**Vimentin promoter sequence**

Vimentin promoter sequence as characterized by the1.5-kb fragment of the human vimentin 5’-upstream region (4) from UCSC genome browser was importer into VectorBuilder to generate Vimentin-promoter-mCherry construct.

5’ - agaatgtagcctgttgcaaagcttccctgggaatacaaagactcctatta

tttctgtccagagccaccacatttgccagaatgtctgtatctacattcca

tagtgaagcccatagaaaccattaataccccttagccaaggggacagatg

gctccttttcacccttattagatcacaaaatagttgtgcatagtgatact

tccatcgaactttaatgacacaatagaatttgcaaagaagaaaagaaact

tgctggctccattccagttggaaactaagttttcttatcttccaacacgt

tttccattttaaactgcagggcataattgcgctttttgtggccttgtaaa

aattaagcaacttgactttggcaaaccacaatctaaaattgtaaccgttc

gaaaccagaaggtcaatgtgttgcaaaactgtgcaacatttgggaaacaa

agtaaaaagttaaaagatttttctttatgtcccaaggaactgcttagaaa

tcattcaaggatcgcttgaacccgggaggcggaggatgcagtgagccaag

atctcgccactgcactccagcctgggcgacagagtaagactccgtctcaa

aaaaaaaaaaaaaatcgctcaaggggttttattaatatttttctaatcat

gagaagctccaacctgtggtcttcccgccactagagggctttacgcgcag

ccctgaagttttgcactttttagacgtgagttaagcaacttttagaattg

ctcgtgggttgtgttgggtggggtcaggcggctgcgagtgggaagagagt

ctacaaacctgtctgaaagattgcagggtgttttcggcttcctctctctg

aagcatctcctcctgcaatctgaaacagttgcgcattaaccaaaggggaa

cgaggaaaccgccaggggcggggcggccacacccaaacaccacgtattcg

ctctgcgcctccagagcagctgcgccacctccgcgcaactagcgcctgca

gctttcagacctgctgccctcggcaggtttcagcgggacttcgggcaccg

cgcagaggggcaatcacggtggccgcacagacctacaggacagccccgcc

atccccgcccccgagggaccatccctttgtctcgctccctccaccgcctt

cccctccttccttctcccgcccggtgattggcagcctgccagaaggggca

ggaaactttctgaaagtttggaggactggctctcattgtgcccaagggcc

ttcaactgcacacaaagtggtagttttaagaaatctgtaacttgaaacgg

agcgtccttgggcaatgtgtggggacagaggaggaaatgcgaactgcaag

gtctgggttctgggcggggctgcgccaccacagccacagccccgctctcc

gctgtctccctggcaggcctccccccggccacatccccaagggcgtggca

gctctcgcgggggccgggggggcgttcccctctccctccagccccctccc – 3'

**RNA Sequencing Analysis**

Empty vector HT-29 cells were incubated alone or with LAD2 cells for 12 hours, with N=3 independent biological replicates. RNA extraction was performed using the RNAeasy mini kit (Qiagen, 74104) for HT-29 cell samples and RNAeasy micro kit (Qiagen, 74004) for LAD2 cells samples as per manufacture’s protocol. Libraries were generated for sequencing using the NEBNext Ultra II DNA Library Prep kit for Illumina (NEB, E7645) as per the manufacturer’s protocol, followed by sequencing. Sequencing read quality control for all samples was assessed with FastQC (v0.11.5). For RNA-seq analysis, read alignment was performed using STAR (5) (v2.6.1a) against the hg38 reference genome, raw read counts for genes were obtained with Rsubread (6) (v1.24.2) featureCounts, and DESeq2 (7) (v1.46.0) was used for differential expression analysis. Gene Set Enrichment Analysis using Gene Ontology database (8) were run with gene set enrichment analysis (9) (v3.12.0). All scripts used for the above analysis are available upon request.

**Supplementary References**

1. Roy A, Libard S, Weishaupt H, Gustavsson I, Uhrbom L, Hesselager G, et al. Mast cell infiltration in human brain metastases modulates the microenvironment and contributes to the metastatic potential. Front Oncol. 2017 Jun 2;7(JUN).

2. Kuehn HS, Radinger M, Gilfillan AM. Measuring mast cell mediator release. Curr Protoc Immunol. 2010 Nov;Chapter 7:Unit7.38.

3. Dame MK, Attili D, McClintock SD, Dedhia PH, Ouillette P, Hardt O, et al. Identification, isolation and characterization of human LGR5-positive colon adenoma cells. Development. 2018 Mar 15;145(6).

4. Gilles C, Polette M, Zahm JM, Tournier JM, Volders L, Foidart JM, et al. Vimentin contributes to human mammary epithelial cell migration. J Cell Sci. 1999 Dec 15;112(24):4615–25.

5. Dobin A, Davis CA, Schlesinger F, Drenkow J, Zaleski C, Jha S, et al. STAR: ultrafast universal RNA-seq aligner. Bioinformatics. 2013 Jan 1;29(1):15–21.

6. Liao Y, Smyth GK, Shi W. The R package Rsubread is easier, faster, cheaper and better for alignment and quantification of RNA sequencing reads. Nucleic Acids Res. 2019 May 7;47(8):e47–e47.

7. Love MI, Huber W, Anders S. Moderated estimation of fold change and dispersion for RNA-seq data with DESeq2. Genome Biol. 2014 Dec 5;15(12):550.

8. Harris MA, Clark J, Ireland A, Lomax J, Ashburner M, Foulger R, et al. The Gene Ontology (GO) database and informatics resource. Nucleic Acids Res. 2004 Jan 1;32(Database issue):D258-61.

9. Subramanian A, Tamayo P, Mootha VK, Mukherjee S, Ebert BL, Gillette MA, et al. Gene set enrichment analysis: A knowledge-based approach for interpreting genome-wide expression profiles. Proceedings of the National Academy of Sciences. 2005 Oct 25;102(43):15545–50.
